# Osteopathic Considerations for Peripheral Neuropathy Due to Concomitant Diffuse Idiopathic Skeletal Hyperostosis Syndrome and Lumbar Epidural Lipomatosis: Case Report

**DOI:** 10.2196/14607

**Published:** 2019-11-20

**Authors:** Justin Chin, Bina Kviatkovsky, Christine Lomiguen

**Affiliations:** 1 Touro College of Osteopathic Medicine New York, NY United States; 2 Staten Island University Hospital Northwell Health Staten Island, NY United States; 3 Lake Erie College of Osteopathic Medicine Erie, PA United States

**Keywords:** diffuse idiopathic skeletal hyperostosis syndrome, epidural lipamtosis, ostepathic medicine

## Abstract

**Background:**

Diffuse idiopathic skeletal hyperostosis (DISH) syndrome and lumbar epidural lipomatosis are relatively asymptomatic neurological conditions, with findings often seen incidentally on radiological studies.

**Objective:**

The aim of this paper is to present unique findings of concomitant, symptomatic DISH syndrome and lumbar epidural lipomatosis and to discuss the osteopathic diagnosis and treatment implications.

**Methods:**

Concomitant, symptomatic variants are rare and present challenges to treatment and management, as seen with a 60-year-old African American woman who presented with worsening disequilibrium and new-onset bilateral fingertip numbness. Past medical history was significant for alcohol abuse disorder, hypertension, hyperlipidemia, and multiple episodes of self-resolving vertigo and lower extremity neuropathy.

**Results:**

The patient was referred to the neurology department for stroke workup, which was negative. Osteopathic structural exam revealed thoracolumbar and sacral dysfunctions. Magnetic resonance imaging revealed findings consistent with thoracic DISH syndrome and lumbar epidural lipomatosis in the areas of somatic dysfunctions.

**Conclusions:**

Due to minimal information on osteopathic manipulative treatment in rare neurological diseases, only gentle techniques of myofascial release, balanced ligamentous tension, and muscle energy were performed with resultant minimal improvement, thus highlighting the necessity for better guidelines and further research.

## Introduction

Neurological symptoms are notoriously nonspecific, with differentials ranging from primary causes such as a tumor to secondary causes of dysfunction in other body systems. Further complicating the diagnosis, one-third of all neurological patients have findings that can only be partially or not at all explained by a discrete, organic disease [1]. Psychosocial and other external factors also play a role in neurological symptoms and disease manifestation [1,2]. With considerable overlap in epidemiology, pathogenesis, and treatment options for various neurological diseases and syndromes of exclusion, patients often spend much of their time searching for a panacea, leading to endless frustration and delayed diagnosis/treatment.

History elicitation is difficult, as patients often lack the ability to distinguish the subtle differences that separate one disease from another. Studies have shown that patients frequently give unclear, inconsistent, and unreliable answers, especially in acute care/emergency room settings [1,2]. Osteopathic physicians are trained to assess the person as a unit of body, mind, and spirit. Osteopathic manipulative treatment (OMT) is based on the understanding of this relationship in helping the body return to a state of homeostasis and health maintenance. Here, we present a complicated case of dizziness and peripheral neuropathy with associated osteopathic findings unresponsive to OMT and subsequent possible etiologies elucidated on radiological workup.

With no reports of concomitant, symptomatic disseminated idiopathic skeletal hyperostosis (DISH) syndrome and lumbar epidural lipomatosis, the aim of this paper is to present these unique findings and discuss the osteopathic diagnosis and treatment implications.

## Methods

### Overview

A 60-year-old African American female with a past medical history of alcohol abuse, controlled hypertension, and hyperlipidemia presented to the emergency department with a 1-week history of worsening disequilibrium and new bilateral upper extremity fingertip numbness after a fall.

### Case Presentation

In the emergency room, she denied any noticeable trauma, loss of consciousness, headache, diplopia, hearing loss, tinnitus, nausea, or vomiting at the time. Previously, she had vertiginous symptoms for several years, with minimal improvement on meclizine and an inconclusive workup with her primary care physician. Separately, she also reported a long history of self-resolving lower extremity numbness/neuropathy that she had associated with her previous occupation as a housekeeper, which required her to frequently be on her knees and feet. At the time of admission, she complained that her lower extremities were now constantly numb, with new intermittent burning (7/10 on the Numeric Pain Scale), nonradiating pain, which limited her ability to walk. Noncontrast head computed tomography was performed for stroke concerns, with subsequent results showing no acute intracranial pathology other than mild, chronic microvascular changes. She was referred to the neurology department for further workup.

## Results

A physical exam at admission revealed nonacute distress, with vitals within normal limits. The neurological exam was positive for right eye horizontal and vertical nystagmus, lower extremity motor weakness, and subjective bilateral sensory paresthesia in dermatomes T10-S2. The patient was able to distinguish soft versus sharp touch, but reported that it was a blunted sensation when compared to dermatomes above T10. Upper extremity motor and sensory exams were normal, with apparent resolution of her bilateral fingertip numbness. Orthostatic exam showed all values within normal limits and negative improvement of symptoms with the Dix-Hallpike and Epley maneuvers. Complete blood count, comprehensive metabolic panel, autoimmune panel, vitamin B12, and thyroid panels were within standard ranges.

Osteopathic structural exam revealed numerous somatic dysfunctions: occipital-atlas joint flexed-rotated right-side bent left, atlantoaxial joint rotated right, C4 vertebra flexed-rotated right and side bent right, T5-T9 vertebral in neutral rotation left and side bent right, T12-L2 vertebra in neutral rotation right and side bent left, right-on-right sacral torsion, and left posteriorly rotated innominate. No appreciable viscerosomatic reflexes were visualized. Upper and lower extremity somatic dysfunctions were negligible. Gentle myofascial release, balanced ligamentous tension, and muscle energy were selected and applied to the thoracolumbar, sacral, and innominate somatic dysfunctions with minimal improvement in symptoms. Cervical treatment was deferred due to patient request. Reassessment showed persistent thoracolumbar dysfunctions. Further trials of OMT were deferred on the patient’s request.

**Figure 1:**
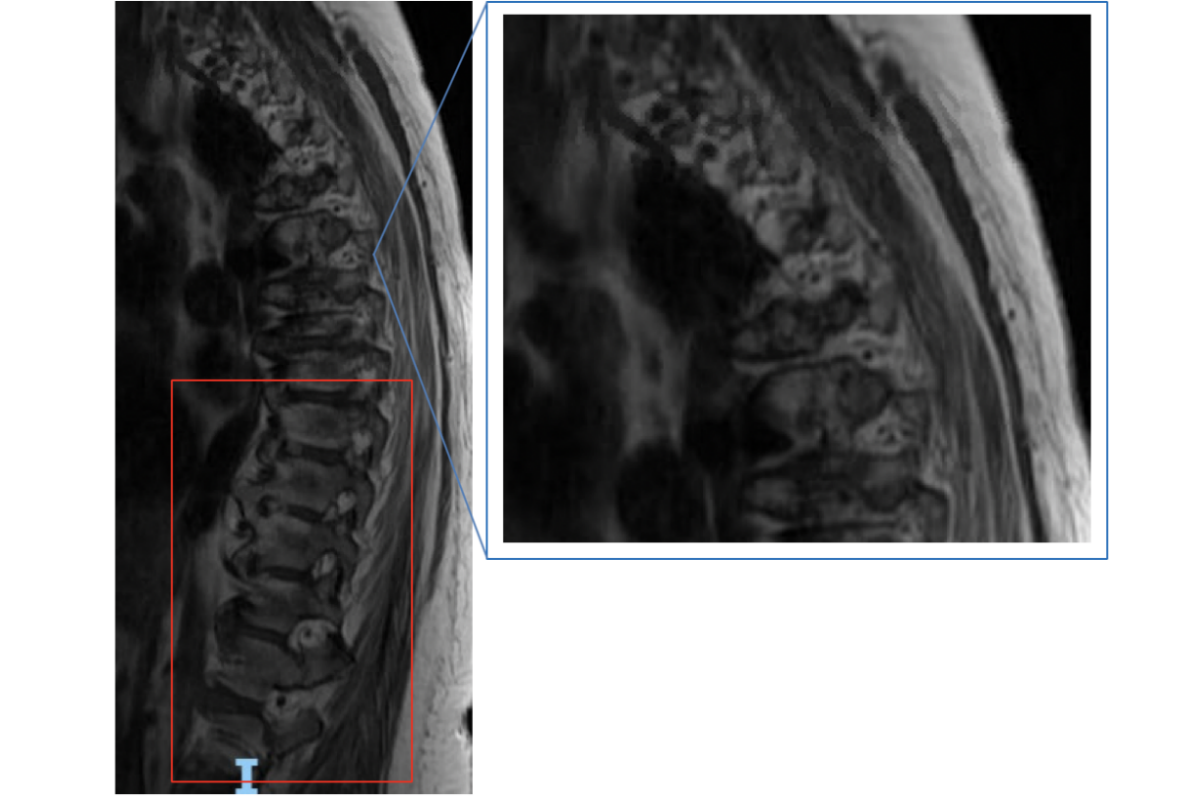
T1-weighted sagittal magnetic resonance imaging of the cervical and thoracic spine revealing C4-C5 osteophyte complex with associated C2-C7 spinal cord compression of the thecal sac secondary to disc osteophyte complexes (blue box) and disseminated idiopathic skeletal hyperostosis syndrome throughout the thoracic spine, particularly at the levels of T5-T9 (red box). Orientation: I indicates inferior.

**Figure 2:**
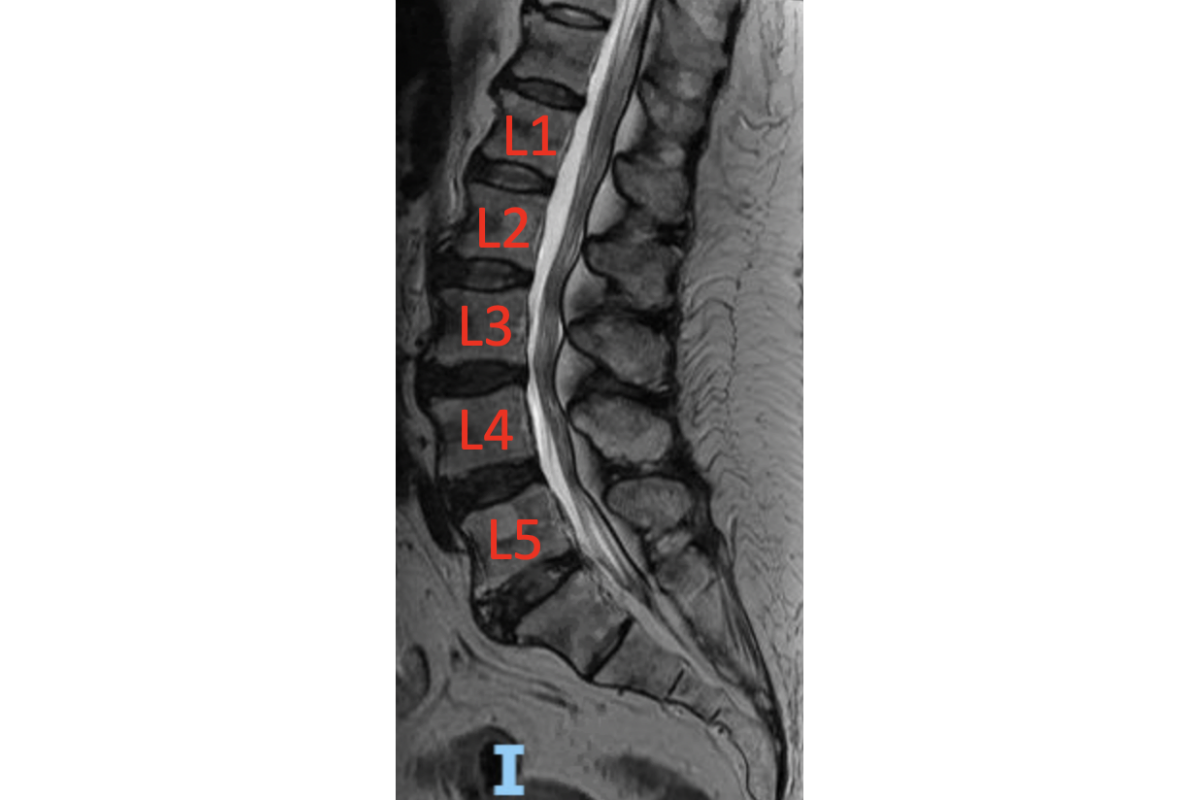
T2-weighted sagittal magnetic resonance imaging of the lumbar spine revealing moderate stenosis of L2-L3 with epidural lipomatosis at L3-L4. Orientation: I for inferior.

Further workup on computed tomography angiogram revealed a 2.5 × 1.5 mm saccular aneurysm from the anterior communicating artery, hypoplastic A1 segment of the right anterior cerebral artery, small vertebrobasilar circulation, and dominant anterior circulation. Magnetic resonance imaging (MRI) of the cervical and thoracic spine were motion limited, but revealed a C4-C5 osteophyte complex with associated C2-C7 spinal cord compression of the thecal sec secondary to disc osteophyte complexes and disseminated idiopathic skeletal hyperostosis throughout the thoracic spine (Figure 1). MRI of the lumbar spine revealed moderate stenosis of L2-L3 with epidural lipomatosis at L3-L4 (Figure 2). Endovascular and neurosurgical teams were consulted, but the patient declined surgical intervention. Conservative management and supportive care were discussed, and she elected for physical therapy and outpatient nerve conduction studies and electromyography. OMT was also discussed as an alternative method to addressing chronic symptoms.

## Discussion

First described in 1975, spinal epidural lipomatosis (SEL) is rare condition, wherein there is a hypertrophy of adipose tissue in the spinal epidural space that results in compression of nerves in the affected region [3]. SEL occurs in 1 of every 40 patients, with presentations ranging widely from asymptomatic to cauda equina syndrome/permanent nerve damage [4,5]. Although SEL can occur throughout the spinal cord, the majority of cases have been documented in the thoracic and lumbosacral region, with the rarest described in the cervical region [6,7]. The exact pathophysiology of SEL is unknown; however, various associations have been made, depending on the region. Exogenous long-term steroid use and male gender have been associated with thoracic SEL in over 75% of the reported cases [3,7]. In comparison, cervical and lumbosacral SEL are largely idiopathic and incidentally seen on MRI [7]. Other possible associations/causes include Cushing disease, Cushing syndrome, obesity, hypothyroidism, and pituitary adenoma [8,9]. Treatment options for symptomatic SEL range from conservative treatment of dietary changes, weight loss, and long-term steroid weaning to decompressive laminectomy and adipose resection [3,10]. Although studies have shown equal efficacy, surgical intervention is generally reserved for failed conservative management, as there are considerable complications and morbidity associated with the postoperative management of concomitant medical problems [9,10].

DISH syndrome is the abnormal calcification of ligaments or bone formation in the axial or appendicular skeleton [11]. Commonly diagnosed using criteria established in 1976, its alternate names are Forestier disease, senile ankylosing spondylosis, and ankylosing hyperostosis [11,12]. This condition involves abnormal calcification of either the anterior or posterior longitudinal ligament of the spine, with the thoracic region being the most common [12]. Peripherally, it is characterized by calcification and hyperossification of entheses or sites where ligaments/tendons attach to bone [13]. The incidence and prevalence of DISH syndrome are largely unknown and underreported due to its asymptomatic nature [14,15]. Symptomatic manifestation is variable, ranging widely from monoarticular synovitis to mass effect airway obstruction. Similar to SEL, the pathogenesis of DISH syndrome is unclear, with mechanical factors, genetics, environmental exposures, and metabolic/dietary conditions as proposed mechanisms or having possible associations [13,16]. Limited research exists on treatment, with the majority pursuing conservative management, analgesics, physical therapy, or a combination of the aforementioned [15]. In extremely rare cases of extensive calcification or if osteophyte formation is causing severe and focal symptoms, surgical intervention with excision or resection of bony tissue may be warranted [16].

On reviewing the literature, we did not find any reports of patients with symptomatic concomitant lumbar SEL and thoracic DISH syndrome. In our case, the patient had a complicated clinical picture, with multiple comorbidities and disease associations with lumbar SEL and DISH syndrome, such as chronic alcohol abuse, hypertension, hyperlipidemia, and obesity. Although such findings supported a diagnosis of symptomatic lumbar SEL and DISH syndrome, definitive diagnosis and treatment of the patient’s complaints remained elusive, as there is a paucity of research regarding simultaneous disease presentation. Her CT angiogram findings and alcohol abuse disorder history may also have played a role in her vertiginous symptoms. In both lumbar SEL and DISH syndrome, studies have explored the difficulty in creating a comprehensive, conservative management plan due to variability in symptom manifestation and severity. With this unpredictable expression, OMT has been proven to be an effective modality for treating similar intractable pain/discomfort syndromes that are refractory to established management options [17]. Treatments of both DISH- and SEL-affected areas were performed together, as minimal differences for standard treatments exist between the two conditions [3,15].

Coupled with the osteopathic philosophy of seeing the patient as a sum of mind, body, and spirit, greater research needs to be conducted on application of osteopathic treatment to rarer diseases and syndromes. Although somatic dysfunction can be an indicator of an underlying pathology, there is conflicting and limited data on osteopathic diagnosis and subsequent treatment in rarer diseases [18]. Furthermore, while general guidelines exist on the use of direct versus indirect techniques, more studies are needed to categorize the efficacy of said techniques on rarer diseases and the treatment frequency [19]. Greater standardization and blinded trials are needed to be better able to reproduce results and studies [20]. Furthermore, the patient was lost to follow-up after hospital discharge, despite attempts to contact the patient.

Concomitant DISH syndrome and lumbar spinal epidural lipomatosis may present symptomatically with peripheral neuropathy. DISH syndrome and SEL have various disease associations; however, the pathogenesis remains unclear, which hinders the development of treatment options. Case presentation of a patient with these symptoms serves to highlight the complexity and variability of disease presentation. Limited research exists on concomitant DISH syndrome and lumbar SEL as well as the effectiveness of osteopathic management. Further research is required to better understand and develop osteopathic treatment for rare syndromes that are refractory of conservative or traditional management.

## Abbreviations

DISH: diffuse idiopathic skeletal hyperostosis
MRI: magnetic resonance imaging
OMT: osteopathic manipulative treatment
SEL: spinal epidural lipomatosis
